# Accuracy of new Corvis ST parameters for detecting subclinical and clinical keratoconus eyes in a Chinese population

**DOI:** 10.1038/s41598-021-84370-y

**Published:** 2021-03-02

**Authors:** Shengwei Ren, Liyan Xu, Qi Fan, Yuwei Gu, Kaili Yang

**Affiliations:** Henan Provincial People’s Hospital, Henan Eye Hospital, Henan Eye Institute, People’s Hospital of Zhengzhou University, Henan University People’s Hospital, 7 Weiwu Road, Zhengzhou, 450003 Henan People’s Republic of China

**Keywords:** Medical research, Diseases, Eye diseases

## Abstract

This study aimed to compare the values of new corneal visualization Scheimpflug technology (Corvis ST) parameters in normal, subclinical keratoconus (SKC) and keratoconus (KC) eyes, and evaluate the diagnostic ability to distinguish SKC and KC eyes from normal eyes. One-hundred normal, 100 SKC and 100 KC eyes were included in the study. Corvis ST parameters containing dynamic corneal response parameters were measured by one ophthalmologist. The receiver operating characteristic curve was used to evaluate the diagnostic ability of new Corvis ST parameters. The new Corvis ST parameters in KC eyes were different from those in the control and SKC eyes after adjusting for IOP and CCT, and stiffness parameter at the first applanation (SP-A1) and Corvis biomechanical index (CBI) were significantly different between the control and SKC eyes (all *P* < 0.05). The parameter with the highest diagnostic efficiency was SP-A1 (Youden index = 0.40, AUC = 0.753), followed by CBI (Youden index = 0.38, AUC = 0.703), and Integrated Radius (Youden index = 0.33, AUC = 0.668) in diagnosing SKC from control eyes. New Corvis ST parameters in SKC eyes were significantly different from normal control and KC eyes, and could be considered to distinguish SKC and KC eyes from normal eyes.

## Introduction

Keratoconus (KC) is a progressive ectatic corneal disease characterized by corneal thinning and irregular astigmatism^1^. KC usually begins in the second or third decade of life, with a heavy financial burden on the patients and society^2^. Previous studies have reported that the prevalence of KC ranges from 0.17‰ in the United States to 10‰ in Iran, and Asia usually has a higher ratio than Caucasian people^3^. Global consensus on KC proposed that a true unilateral KC does not exist^4^. The contralateral normal eye of a KC patient, also called subclinical KC (SKC) eye and very asymmetrical ectasia, is regarded as the early stage of KC^5,6^. Studying the characteristics of SKC eyes can help to understand the mechanism of KC occurrence and development. Previous studies have reported that the topographic and tomographic parameters of SKC eyes are different from normal eyes, playing a role in diagnosing SKC eyes^7–9^. It has been reported that changes in corneal biomechanics occur earlier than those in the topographic and tomographic maps in KC eyes, resulting in more attention to corneal biomechanics in clinical application^10^.

Corneal visualization Scheimpflug technology (Corvis ST) is a relatively new non-contact tonometer, and obtains dynamic corneal response (DCR) parameters with a rate of 4330 frames/s^11^. With the software updated (number: 1.5r1902), new parameters were gradually used in the clinic^12^. Our previous studies found that new Corvis ST parameters were different between KC and normal control eyes^12,13^. Several studies have also reported that new Corvis ST parameters of SKC eyes are different from normal and KC eyes^14,15^. The diagnostic efficiency of new Corvis ST parameters in diagnosing SKC eyes from normal eyes is inconsistent^14–16^. Kataria et al.^14^ reported that the area under the curve (AUC) of new Corvis ST parameters ranged from 0.512 (Pachy Slope) to 0.775 (Corvis biomechanical index, CBI) in distinguishing SKC patients from normal Indian subjects. Chan et al.^15^ also found that Ambrósio’s relational thickness horizontal (ARTh) and Max Inverse Radius played roles in distinguishing SKC eyes from normal eyes. Furthermore, our previous study showed that abnormal pachymetry distribution is detectable in SKC eyes with normal biomechanics through receiver operating characteristic (ROC) analysis, while the analysis of new Corvis ST parameters of our SKC eyes has not been carried out^5^. In addition, corneal central thickness (CCT) is a fundamental parameter affecting the corneal biomechanical properties. Knowing the association between new Corvis ST parameters and CCT in normal, SKC, and KC eyes could help clinicians make better use of these parameters.

Thus, the current study aimed to compare the new Corvis ST parameter values in normal, SKC, and KC eyes, and further evaluate the ability of new Corvis ST parameters to distinguish SKC and KC eyes from normal eyes.

## Methods

### Study subjects

This prospective study was conducted between September 2018 and January 2020. Clinical KC in current study was diagnosed according to the following criterion: (1) corneal topography revealing an asymmetrical bowtie pattern with or without skewed axes; (2) KC sign on slit-lamp examination, such as localized stromal thinning, conical protrusion, Vogt’s striae, Fleischer’s ring, or anterior stromal scar; (3) Belin Ambrosio enhanced ectasia total deviation index (BAD-D) value > 2.6^1,13^. The SKC eye in the current study was defined by (1) no clear evidence of KC in one eye; (2) the contralateral eye diagnosed with clinical KC eye^17^. Volunteers were recruited in the control group with (1) spherical equivalent < -8.00 diopters (D), astigmatism < 2.00 D, corrected distance visual acuity (CDVA) ≥ 0.8; (2) normal corneal topography (the central region is generally steeper, gradually flattening to the periphery) and BAD-D value < 1.6^1^. The exclusion criteria consisted of eyes with a history of wearing soft contact lens within two weeks, wearing rigid contact lens within four weeks, eyes with an anterior stromal scar, other ocular diseases, ocular trauma, and ocular surgery. Finally, 100 KC eyes (19 unilateral KC and 77 bilateral KC patients), 100 SKC eyes (100 unilateral KC patients), and 100 control eyes (100 normal subjects) were included.

### Examinations

**Figure 1:**
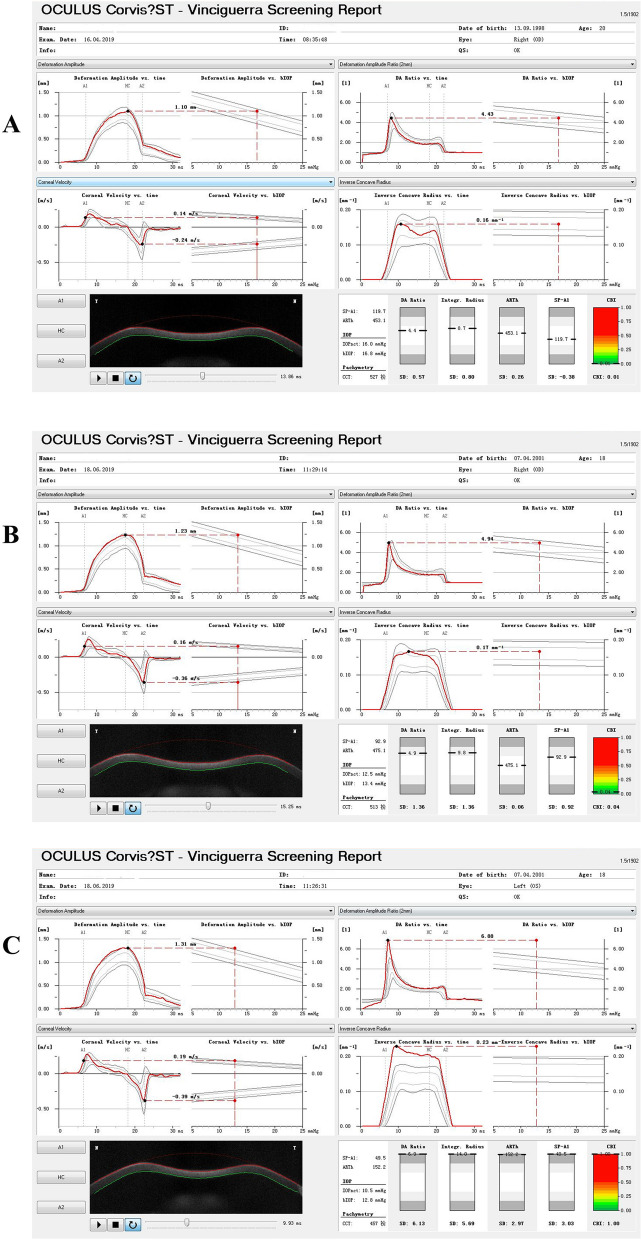
New parameters measured by Corvis ST. (**A**) control eyes; (**B**) SKC eyes; (**C**) KC eyes.

The Corvis ST instrument takes Scheimpflug images of the anterior segment at a rate of 4330 frames/s and collects parameters during the first applanation, highest concavity, and second applanation phases (time, velocity, deflection amplitude, and deflection length)^10^. Using the updated software (software number: 1.5r1902), new parameters were added, such as Max Inverse Radius (the maximum value of the radius of curvature during the concave phase of the deformation)^18^, deformation amplitude (DA) Ratio Max (1 mm) and DA Ratio Max (2 mm) (the maximum ratio of DA measured at 1 or 2 mm from the center of the cornea, with higher values describing less resistance to corneal deformation^15^), Pachy Slope (changes of corneal thickness from the corneal center to the periphery, averagely determined at 2.5 mm from the apex^19^), ARTh (a parameter calculated by the division of the thinnest corneal thickness and pachy metric progression index^12^), Integrated Radius (integrated area under the curve of the Inverse Radius^20^), stiffness parameter at the first applanation (SP-A1, a resultant pressure of the first applanation calculated as the adjusted pressure minus intraocular pressure (IOP) divided by the deflection amplitude^21^) and CBI (a combined parameter based on a logistic regression formula^10^). Corvis ST records information concerning the cornea’s stiffness data (SP-A1) and viscoelastic properties [DA Ratio Max (2 mm), DA Ratio Max (1 mm), and Integrated Radius] throughout the deformation process^22^. The new Corvis ST parameters of control, SKC, and KC eyes are shown in Fig. 1. Three repeated measurements with QS showing OK were conducted by one ophthalmologist between 9:00 and 17:00, and the average measurements were analyzed in the current study.

In addition, all the participants received a slit-lamp examination and Pentacam HR measurements to collect steep keratometric (Ks), flat keratometric (Kf), and mean keratometric (Kmean), CCT, Astigmatism F, Axis F(steep), inferior-superior difference value (I–S) and BAD-D. Furthermore, CDVA, tomographic and biomechanical index (TBI) and KISA%^23^ values were recorded in the current study.

### Statistical analysis

The distribution of the variables was tested through the Kolmogorov–Smirnov test. Normally distributed data were presented in mean ± SD and analyzed by ANOVA, and non-normally distributed data were presented in median (interquartile range, IQR) and analyzed by Kruskal–Wallis test. Linear mixed-effect models were constructed, adjusting for the effect of IOP and CCT on corneal deformation response parameters, and further comparisons of the least significant difference(LSD) were carried out. The ROC curve and Delong test were used to evaluate the diagnostic ability of new Corvis ST parameters for distinguishing KC and SKC eyes from normal control eyes. Pearson’s or Spearman’s correlation analysis was conducted to investigate the association between CCT and new Corvis ST parameters. All the statistical analyses of the survey data were performed using SPSS 23.0 software package and MedCalc 15.2.2 software, and a *P* < 0.05 (two-tailed) was considered as statistically significant difference.

### Ethics approval and informed consent

This study was conducted following the Declaration of Helsinki guidelines and approved by the Institutional Review Board of Henan Eye Hospital [ethical approval number: HNEECKY-2019 (5)]. Written informed consent was obtained from each patient.

## Results

### Characteristics of basic parameters

The basic parameters of control, SKC and KC eyes were presented in Table 1. The mean ages were 23.36 ± 4.76 years, 22.79 ± 5.78 years, and 23.44 ± 5.36 years for the control, SKC, and KC patients, respectively (*P* = 0.642). The KC eyes exhibited weaker CDVA, and higher Ks, Kf, Kmean, I–S, astigmatism F and KISA% values, and lower IOP and CCT values compared to the control and SKC eyes (all *P* < 0.05). The values of BAD-D and TBI in SKC eyes were higher than the control eyes (all *P* < 0.05).

**Table 1:** Comparisons of basic parameters among control, SKC and KC eyes.

Parameters	Control (N = 100)	SKC (N = 100)	KC (N = 100)	P*	*P*^#1^	*P*^#2^	*P*^#3^
CDVA (LogMAR), median (IQR)	0.00 (0.00)	0.00 (0.00)	0.22 (0.30)	< 0.001	0.454	< 0.001	< 0.001
IOP (mmHg), median (IQR)	16.00 (2.50)	14.50 (2.00)	13.00 (3.40)	< 0.001	< 0.001	< 0.001	< 0.001
CCT (mm), mean ± SD	553.34 ± 30.10	531.44 ± 25.94	486.58 ± 34.39	< 0.001	< 0.001	< 0.001	< 0.001
Ks (D), mean ± SD	43.65 ± 1.46	43.65 ± 1.48	49.43 ± 4.25	< 0.001	1.000	< 0.001	< 0.001
Kf (D), median (IQR)	42.70 (1.67)	42.40 (2.07)	45.20 (3.98)	< 0.001	1.000	< 0.001	< 0.001
Kmean (D), median (IQR)	43.30 (1.75)	42.96 (2.10)	46.90 (4.48)	< 0.001	1.000	< 0.001	< 0.001
I-S (D), median (IQR)	0.27 (0.91)	0.27 (0.93)	4.22 (4.69)	< 0.001	0.781	< 0.001	< 0.001
Astigmatism F, median (IQR)	1.00 (0.90)	1.00 (0.90)	3.05 (3.00)	< 0.001	0.947	< 0.001	< 0.001
Axis F (steep), median (IQR)	90.10 (12.50)	92.90 (28.50)	89.05 (46.80)	0.863	0.589	0.823	0.743
BAD-D, median (IQR)	0.88 (0.66)	1.55 (1.13)	8.15 (5.00)	< 0.001	0.034	< 0.001	< 0.001
TBI, median (IQR)	0.13 (0.21)	0.34 (0.44)	1.00 (0.00)	< 0.001	0.031	0.029	0.031
KISA%, median (IQR)	12.15 (21.74)	12.68 (32.04)	490.01 (1230.31)	< 0.001	0.972	< 0.001	< 0.001

*SKC* Subclinical keratoconus, *KC* Keratoconus, *CDVA* Corrected distance visual acuity, *IOP* Intraocular pressure, *CCT* Central corneal thickness, *Ks* Steep keratometric, *Kf* Flat keratometric, *Kmean* Mean keratometric, *I–S* Inferior-superior difference value, *BAD-D* Belin Ambrosio enhanced ectasia total deviation index, *TBI* tomographic and biomechanical parameters.

*ANOVA or Kruskal–Wallis test, ^#1^control versus SKC; ^#2^control versus KC; ^#3^SKC versus KC.

### Characteristics of new Corvis ST parameters

**Figure 2:**
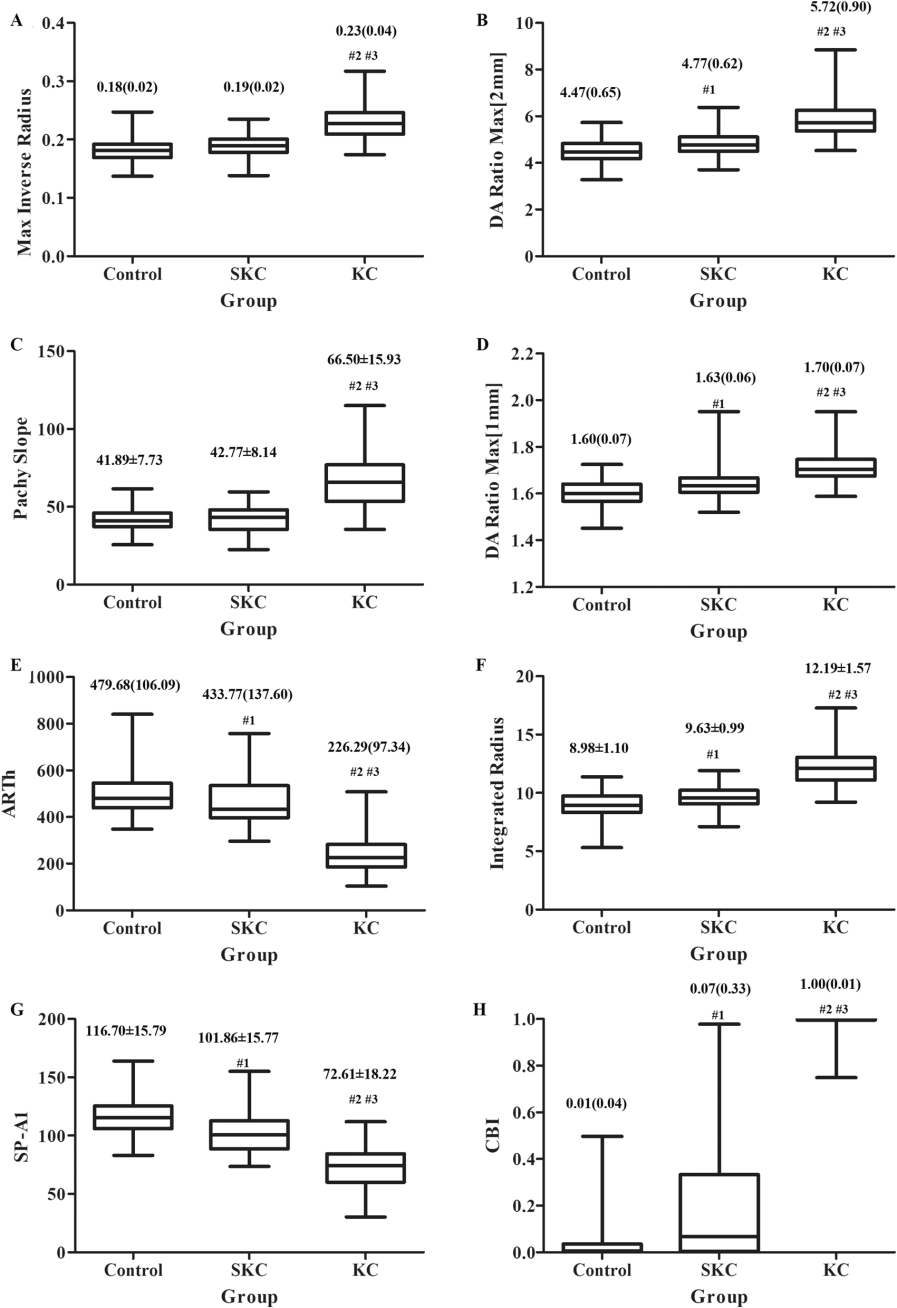
The box plot for new variables measured by Corvis ST (^#1^, *P* < 0.05 for SKC vs control; ^#2^, *P* < 0.05 for KC vs control; ^#3^, *P* < 0.05 for KC vs SKC). (**A**) Max inverse radius; (**B**) DA ratio max (2 mm); C, Pachy slope; (**D**) DA ratio max (1 mm); (**E**) ARTh; (**F**) integrated radius; (**G**) SP-A1; H, CBI.

The new Corvis ST parameters in the three groups were presented in Fig. 2. The Max Inverse Radius, DA Ratio Max (2 mm), Pachy Slope, DA Ratio Max (1 mm), ARTh, Integrated Radius, SP-A1 and CBI were statistically different in three groups (all *P* < 0.001). After adjusting for IOP and CCT, these parameters in KC eyes were different from those in the control and SKC eyes (all *P* < 0.001), and SP-A1 and CBI were significantly different between the control and SKC eyes (*P* = 0.011 for SP-A1 and *P* < 0.001 for CBI, Table 2).

**Table 2:** The results of linear mixed-effect model adjusting for IOP and CCT.

Mean estimates values	Control (N = 100)	SKC (N = 100)	KC (N = 100)	P*	*P*^#1^	*P*^#2^	*P*^#3^
Max inverse radius	0.19	0.19	0.22	< 0.001	0.459	< 0.001	< 0.001
DA ratio max (2 mm)	4.85	4.86	5.47	< 0.001	0.937	< 0.001	< 0.001
Pachy slope	40.49	42.38	68.30	< 0.001	0.261	< 0.001	< 0.001
DA ratio max (1 mm)	1.63	1.65	1.69	< 0.001	0.198	< 0.001	< 0.001
ARTh	477.92	459.75	258.93	< 0.001	0.147	< 0.001	< 0.001
Integrated radius	9.65	9.74	11.51	< 0.001	0.550	< 0.001	< 0.001
SP-A1	101.99	98.99	88.77	< 0.001	0.011	< 0.001	< 0.001
CBI	0.09	0.22	0.92	< 0.001	< 0.001	< 0.001	< 0.001

*SKC* Subclinical keratoconus, *KC* Keratoconus, *DA* Deformation amplitude, *ARTh* Ambrósio’s relational thickness horizontal, *bIOP* Biomechanical corrected intraocular pressure, *SP-A1* Stiffness parameter at the first applanation, *CBI* Corvis biomechanical index.

*Linear mixed-effect model; ^#1^control versus SKC; ^#2^control versus KC; ^#3^SKC versus KC.

### ROC curve analyses of new Corvis ST parameters

The accuracy of new Corvis ST parameters in identifying SKC and KC eyes were presented in Table 3. The highest diagnostic index was CBI (Youden index = 0.99, AUC = 0.999), followed by ARTh (Youden index = 0.96, AUC = 0.992), and SP-A1 (Youden index = 0.84, AUC = 0.973) in KC identification from control eyes. In diagnosing KC from SKC eyes, the highest diagnostic index was CBI (Youden index = 0.93, AUC = 0.992), followed by ARTh (Youden index = 0.88, AUC = 0.980), and Integrated Radius (Youden index = 0.72, AUC = 0.932). Furthermore, the highest diagnostic index was SP-A1 (Youden index = 0.40, AUC = 0.753), followed by CBI (Youden index = 0.38, AUC = 0.703), and Integrated Radius (Youden index = 0.33, AUC = 0.668) in SKC identification from control eyes.

**Table 3:** Accuracy of new Corvis ST parameters in identifying SKC and KC eyes.

Parameters	AUC (95%CI)	Youden index	Cut off	Sensitivity (%)	Specificity (%)
**SKC versus control**					
Max inverse radius	0.626 (0.555, 0.693)	0.23	> 0.19	50	73
DA ratio max (2 mm)	0.684 (0.615, 0.748)	0.33	> 4.47	81	52
Pachy slope	0.538 (0.466, 0.608)	0.16	> 44.30	46	70
DA ratio max (1 mm)	0.673 (0.603, 0.737)	0.31	> 1.60	80	51
ARTh	0.618 (0.547, 0.686)	0.30	≤ 434.02	51	79
Integrated radius	0.668 (0.598, 0.733)	0.33	> 8.94	81	52
SP-A1	0.753 (0.687, 0.811)	0.40	≤ 107.30	66	74
CBI	0.703 (0.635, 0.766)	0.38	> 0.05	56	82
**KC versus SKC**					
Max inverse radius	0.889 (0.837, 0.929)	0.65	> 0.20	87	78
DA ratio max (2 mm)	0.903 (0.853, 0.940)	0.69	> 5.29	80	89
Pachy slope	0.908 (0.859, 0.944)	0.69	> 55.49	73	96
DA ratio max (1 mm)	0.843 (0.785, 0.891)	0.61	> 1.67	79	82
ARTh	0.980 (0.949, 0.994)	0.88	≤ 319.83	89	99
Integrated radius	0.932 (0.887, 0.962)	0.72	> 10.86	81	91
SP-A1	0.893 (0.841, 0.932)	0.64	≤ 82.03	72	92
CBI	0.992 (0.967, 0.999)	0.93	> 0.87	97	96
**KC versus control**					
Max inverse radius	0.923 (0.878, 0.956)	0.71	> 0.19	95	76
DA ratio max (2 mm)	0.953 (0.913, 0.978)	0.77	> 5.13	83	94
Pachy slope	0.919 (0.873, 0.953)	0.69	> 48.46	87	82
DA ratio max (1 mm)	0.914 (0.866, 0.949)	0.71	> 1.66	86	85
ARTh	0.992 (0.967, 0.999)	0.96	≤ 364.89	97	99
Integrated radius	0.965 (0.929, 0.986)	0.79	> 10.50	86	93
SP-A1	0.973 (0.939, 0.991)	0.84	≤ 91.64	87	97
CBI	0.999 (0.980, 1.000)	0.99	> 0.50	99	100

*SKC* Subclinical keratoconus, *KC* Keratoconus, *DA* Deformation amplitude, *ARTh* Ambrósio’s relational thickness horizontal, *bIOP* Biomechanical corrected intraocular pressure, *SP-A1* Stiffness parameter at the first applanation, *CBI* Corvis biomechanical index.

**Figure 3:**
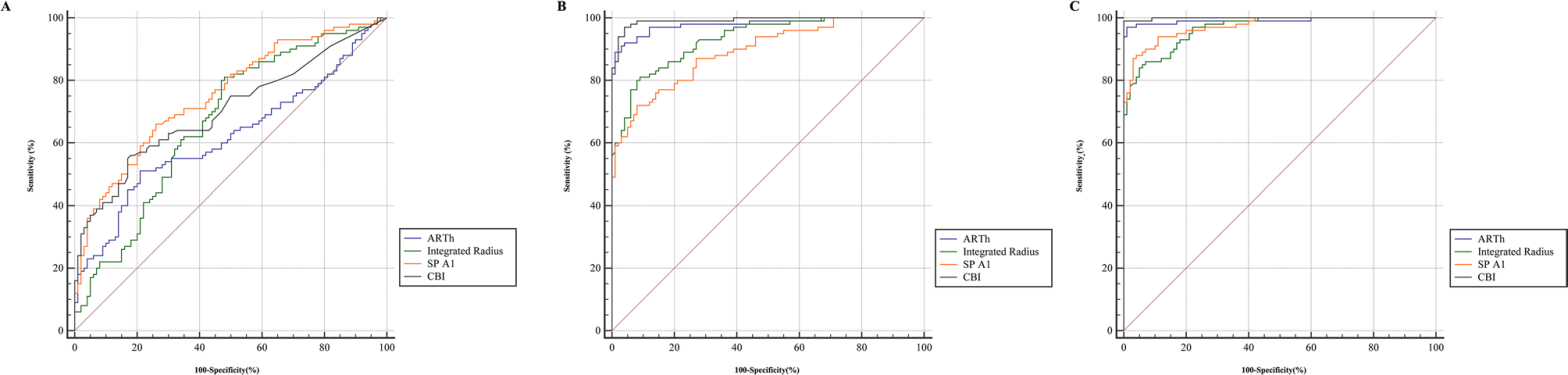
ROC comparisons of ARTh, integrated radius, SP-A1, and CBI in distinguishing SKC and KC eyes. (**A**) SKC versus control; (**B**) KC versus SKC; (**C**) KC versus control.

Further AUC pairwise comparisons of new Corvis ST parameters were presented in Fig. 3 and Table 4. The AUC of CBI was significantly higher than ARTh (Difference = 0.085, *P* < 0.05), and the SP-A1 was significantly higher than Integrated Radius (Difference = 0.085, *P* < 0.05). In contrast, no significant differences were found between Integrated Radius, SP-A1, and CBI in identifying SKC from control eyes (all *P* > 0.05, Fig. 3A). The AUC of CBI was not significantly different from ARTh (all *P* > 0.05), while higher than other parameters in identifying KC eyes from SKC and control eyes (all *P* < 0.05, Fig. 3B,C). The AUC of ARTh was significantly higher than SP-A1 and Integrated Radius in diagnosing KC eyes from SKC and control eyes, while lower than SP-A1 in diagnosing SKC from control eyes (all *P* < 0.05).

**Table 4:** Delong test results for pairwise comparison of the AUCs.

Differences between AUCs	Max inverse radius	DA ratio max (2 mm)	Pachy slope	DA ratio max (1 mm)	ARTh	Integrated radius	SP-A1
**SKC versus control**							
DA ratio max (2 mm)	0.058						
Pachy slope	0.088	0.147*					
DA ratio max (1 mm)	0.047	0.012	0.135*				
ARTh	0.008	0.066	0.081*	0.055			
Integrated radius	0.042	0.016	0.130*	0.005	0.050		
SP-A1	0.127*	0.068*	0.215*	0.080*	0.134*	0.085*	
CBI	0.077	0.019	0.166*	0.031	0.085*	0.035	0.049
**KC versus SKC**							
DA ratio max (2 mm)	0.014						
Pachy slope	0.019	0.005					
DA ratio max (1 mm)	0.046	0.060*	0.065				
ARTh	0.091*	0.077*	0.072	0.137*			
Integrated radius	0.043*	0.029	0.023	0.088*	0.048*		
SP-A1	0.004	0.010	0.016	0.049	0.087*	0.039	
CBI	0.103*	0.089*	0.084*	0.148*	0.012	0.060*	0.099*
**KC versus control**							
DA ratio max (2 mm)	0.029						
Pachy slope	0.004	0.033					
DA ratio max (1 mm)	0.010	0.039*	0.006				
ARTh	0.068*	0.039*	0.072*	0.078*			
Integrated radius	0.042*	0.013	0.046*	0.052*	0.026*		
SP-A1	0.049*	0.020	0.053*	0.059*	0.019*	0.008	
CBI	0.076*	0.047*	0.080*	0.085*	0.008	0.034*	0.026*

*SKC* Subclinical keratoconus, *KC* Keratoconus, *DA* Deformation amplitude, *ARTh* Ambrósio’s relational thickness horizontal, *bIOP* Biomechanical corrected intraocular pressure, *SP-A1* Stiffness parameter at the first applanation, *CBI* Corvis biomechanical index.

*P < 0.05.

### Correlation between CCT and new Corvis ST parameters

**Figure 4:**
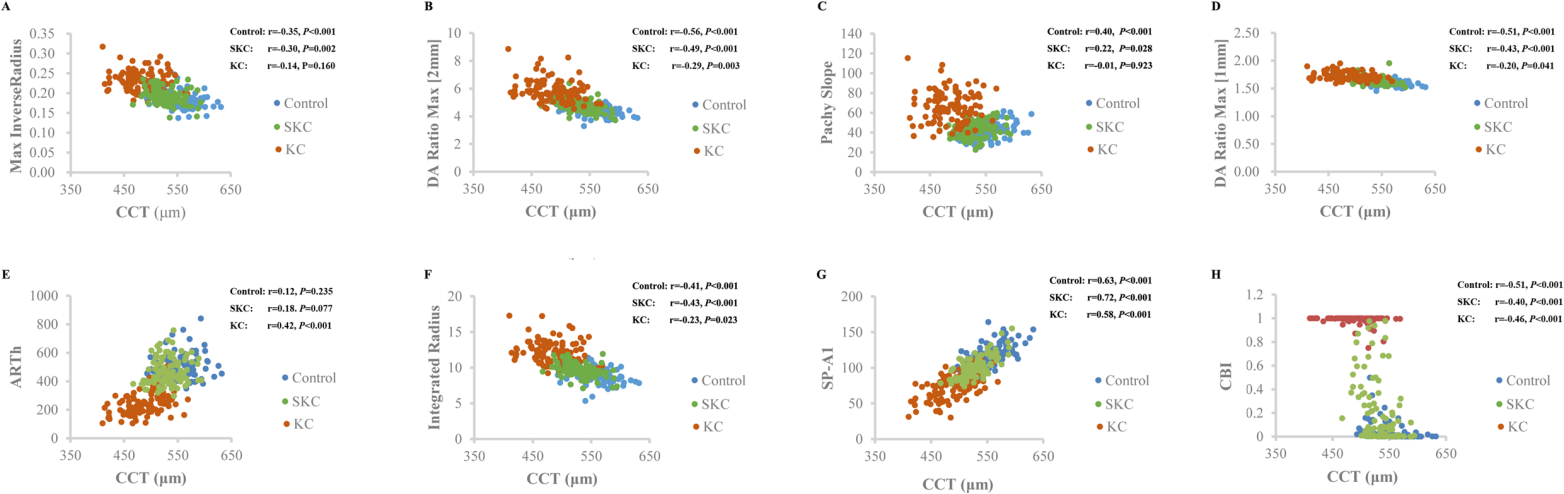
Scatter plot of new Corvis ST parameters and CCT. (**A**) Max inverse radius; (**B**) DA ratio max (2 mm); (**C**) Pachy slope; (**D**) DA ratio max (1 mm); (**E**) ARTh; (**F**) integrated radius; (**G**) SP-A1; (**H**) CBI.

The association between CCT and new Corvis ST parameters was presented in Fig. 4. The Max Inverse Radius (r_Control_ = -0.35, r _SKC_ = -0.30) and Pachy Slope (r _Control_ = 0.40, r _SKC_ = 0.22) were significantly associated with CCT in control and SKC eyes (all *P* < 0.05), while they were not seen in KC eyes (all *P* > 0.05). The DA Ratio Max (2 mm), DA Ratio Max (1 mm), Integrated Radius, SP-A1 and CBI were significantly associated with CCT in control, SKC, and KC eyes (all *P* < 0.05). A significant association was detected between ARTh and CCT in KC eyes (r = 0.42, *P* < 0.001).

## Discussion

Corvis ST is an effective instrument for measuring corneal biomechanics, and the changes in new Corvis ST parameters are of great significance in evaluating KC and SKC eyes. The present study showed that KC had higher values of Max Inverse Radius, DA Ratio Max (2 mm), DA Ratio Max (1 mm), Integrated Radius, and CBI, and lower ARTh and SP-A1 values than control and SKC eyes. The CBI exhibited the highest diagnostic efficiency in distinguishing KC from SKC and control eyes, while the SP-A1 exhibited the highest value in distinguishing SKC eyes from control eyes.

New Corvis ST parameters have been reported to be effective in differentiating KC eyes from normal eyes^10^. The current study showed that KC eyes had higher values of Max Inverse Radius, DA Ratio Max (2 mm), Pachy Slope, DA Ratio Max (1 mm), Integrated Radius and CBI, and lower values of ARTh and SP-A1 than control eyes. The results are consistent to previous studies, indicating that KC eye exhibits a softer cornea, thinner corneal thickness, and greater curvature^14,16^. In addition, the present study showed that the AUC of new Corvis ST parameters in distinguishing KC from control eyes were all > 0.90, consistent with the previous studies^12,14,21,24,25^. CBI is a combined parameter calculated through a formula and was useful in discriminating KC from normal eyes with a cut-off value of 0.5^10^. Steinberg et al.^26^ compared older Corvis ST parameters and CBI, and reported that the concept of KC screening with CBI is effective in differentiating KC from non-KC eyes. The study showed that the AUC of CBI was not significantly different from ARTh, while higher than other new Corvis ST parameters to distinguish KC from control eyes. The results are consistent with Sedaghat MR et al.^21^ and Herber et al.^25^ findings, which validated the clinical application of new Corvis ST parameters in discriminating KC from normal eyes.

Global consensus on KC in 2015 proposed that a true unilateral KC does not exist^4^. Holland et al.^27^ reported that the signs of KC were found in SKC eyes after observation for four years. Li et al.^28^ reported that half of SKC eyes would also develop KC in the following 16 years, with the highest incidence in six years. It is essential for patients and clinicians to be aware of KC development’s probability in the clinically normal eyes of unilateral KC^9^. SKC eye is an ideal model to study the early stages of KC, which shows the earliest detectable signs of the disease. However, there are no specific criteria for SKC eye definition and detection. Previous studies have compared the topographic^5,29^ and tomographic parameters^30,31^, epithelial thickness mapping^7^, and various combinations of indices^23,32^ between SKC eyes and normal eyes, reporting that these parameters could provide references to differentiate SKC from normal eyes for the clinician.

Vinciguerra et al.^16^ reported that an abnormal CBI improved the diagnostic work-up in 12 SKC eyes, Corvis ST parameters used in differentiating SKC eyes from control eyes have gradually attracted attention. Koc et al.^33^ compared DCR parameters obtained from the Corvis ST (A1L, A2L, A1V, A2V, DA Ratio, SP-A1, and CBI) in control, SKC, and KC groups, and the results indicated that biomechanical analysis might be used as a complementary diagnostic method in detecting SKC. Steinberg et al.^34^ reported that older Corvis ST parameters only marginally improved KC screening protocols, and they suggested that newly generated parameters, such as the applanation length level and deflection length level might further improve early KC screening. The current study reported that the Max Inverse Radius, DA Ratio Max (2 mm), DA Ratio Max (1 mm), Integrated Radius, and CBI in SKC eyes were higher than those in normal control eyes, while lower than that in KC eyes. Furthermore, the ARTh and SP-A1 in SKC eyes were lower than those in normal control eyes, while higher than that in KC eyes. The differences of new Corvis ST parameters between the three groups were consistent with previous studies, indicating that the viscoelastic properties and stiffness of SKC eyes have changed, although not up to the level of clinical KC eyes^14,35^. In addition, Chan et al.^15^ reported that the abilities of ARTh and Max Inverse Radius in distinguishing SKC from control eyes were acceptable with AUC values of 0.836 and 0.754, respectively. Compared to Kataria et al.^14^ study, we found the AUC values of Max Inverse Radius, Pachy Slope, Integrated Radius were higher, while DA Ratio Max (2 mm), DA Ratio Max (1 mm), ARTh, SP-A1, and CBI were lower. The discrepancies in the results of these studies could be explained by the differences in SKC criterion. Koc et al.^33^ defined SKC based on Kmean value < 47.20 D, I–S < 1.40 D, KISA% < 60%, with no clinical evidence. Kataria et al.^14^ included patients with frank KC in one eye and a topographically normal contralateral eye and labeled them as SKC. Chan et al.^15^ classified SKC eyes according to whether they exhibited either atypical or suspected topographic findings that did not meet the diagnostic criteria for KC, with average corneal power of 49.00 D or less or HOAs of 1.50 µm or less in either eye or normal topography but obvious KC in the contralateral eye. SP-A1 is an important corneal stiffness parameter defined as adjusted pressure minus IOP and divided by deflection amplitude at the first applanation^21^. The study showed that SP-A1 had the highest accuracy in identifying SKC from control eyes, which suggesting that SP-A1 could be considered in clinical applications in distinguishing SKC from normal eyes. In addition, the study found that the AUC of SP-A1 was significantly higher than that of Integrated Radius in distinguishing SKC from control eyes, similar to Sedaghat MR et al.^21^ findings. However, no study is available on the AUC comparisons of other new Corvis ST parameters. Since research into new Corvis ST parameters of SKC eyes is still limited, further large and multi-center studies are on an absolute necessity.

A previous study found that the biomechanical deterioration and thinning of cornea synchronize with one another throughout the progression of KC^36^. Heber et al.^25^ reported the thinnest corneal thickness was associated with Max Inverse Radius, DA Ratio Max (2 mm), DA Ratio Max (1 mm), Integrated Radius and SP-A1 in healthy and KC eyes through regression analysis. As a parameter affecting the corneal biomechanics in normal eyes, CCT was found to be related with DA Ratio Max (2 mm), DA Ratio Max (1 mm), Integrated Radius, ARTh, SP-A1, and CBI in KC eyes, consistent with Kataria et al.^14^ study. Furthermore, CCT was significantly associated with Max Inverse Radius and Pachy Slope in SKC eyes, not in KC eyes. It could be speculated that the stiffness in the normal cornea mostly arises from layers of collagen lamellae^37^. The breaks in Bowman’s layer, reduced cross-links, and atypical organization of collagen fibrils might be responsible for corneal weakness; then, corneal thickness’s contribution to stiffness would increase^38^. Furthermore, previous study has reported that the corneal thinning in KC eyes decreases the biomechanical properties, resulting in focal weakening of the cornea; a decrease in corneal properties would further thin the cornea^39^.

KC is a localized disease that usually progresses in the vertical meridian, while the Corvis ST just acquires the deformation of the horizontal meridian. The current study limited conducted an analysis of new Corvis ST parameters in differentiating KC, SKC, and normal eyes. A previous study showed that combined use of tomographic and biomechanical parameters resulted in a higher capability to differentiate normal and SKC eyes when compared to analysis alone^6,15,17,40^. Thus, further studies would be conducted to explore the identification ability of comprehensive analysis including tomographic and biomechanical parameters in future clinical applications. Besides, the SKC eyes in the current study were from contralateral eyes of patients with KC, and the unilateral or bilateral SKC (meaning the other eye is not KC) that would develop into KC in the future were not evaluated. Identifying these unilateral or bilateral SKC eyes is a clinical challenge, and further research is recommended in later.

In conclusion, the present study indicated that new Corvis ST parameters of SKC eyes were different from normal control and KC eyes, and could help differentiate KC and SKC eyes from normal eyes in clinical applications.

## Data Availability

All relevant data are included in the papers. Contact to Dr. Shengwei Ren (shengweiren1984@163.com) for additional information regarding data access.
